# Supplementary figures and images for: How sure are you? A web-based application to confront imperfect detection of respiratory pathogens in bighorn sheep
Source: PLoS One. 2020 Sep 8;15(9):e0237309. doi: 10.1371/journal.pone.0237309 (PMC7478830; doi:10.1371/journal.pone.0237309)

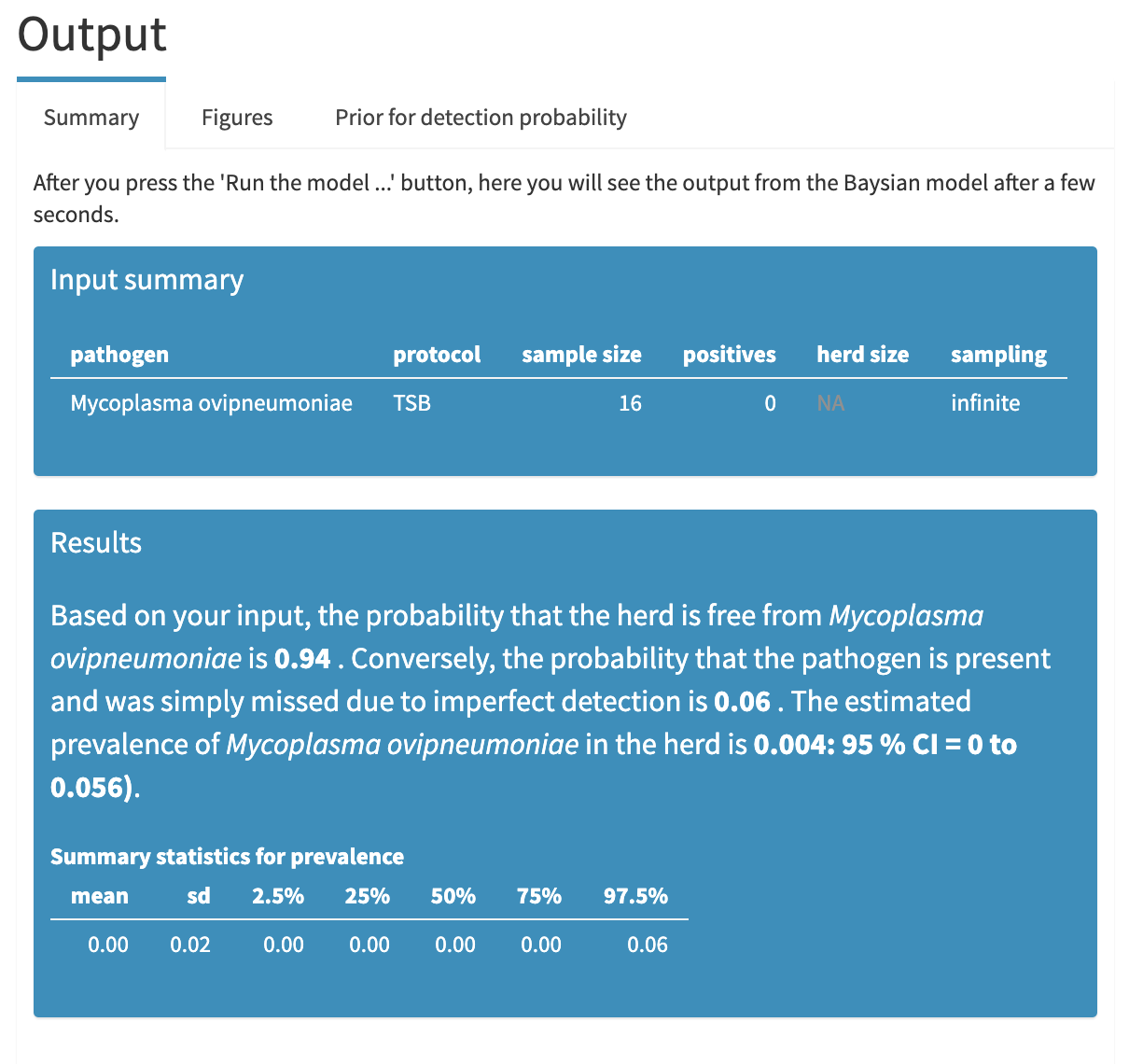

Supplement: S1 Fig — In this specific example, 0 out of 16 animals in the Petty Creek herd (2015–2016) tested positive for Mycoplasma ovipneumoniae using the TSB-PCR protocol with 2 swabs per animal. (TIF) [file pone.0237309.s001.tif]

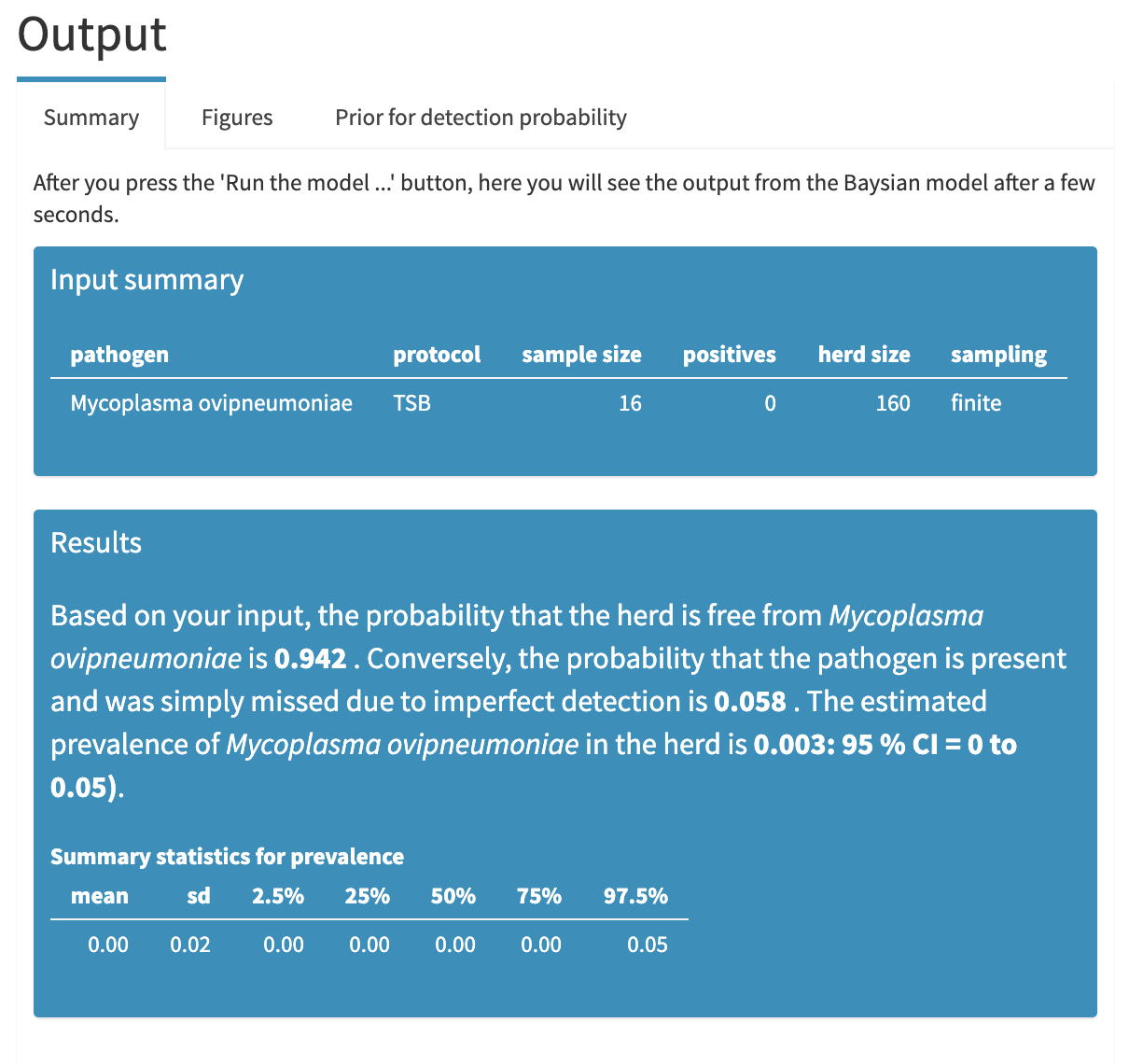

Supplement: S2 Fig — In this specific example, 0 out of 16 animals in the Petty Creek herd (2015–2016) tested positive for Mycoplasma ovipneumoniae using the TSB-PCR protocol with 2 swabs per animal. These results agree closely with those that assumed a finite population (S1 Fig). (TIF) [file pone.0237309.s002.tif]

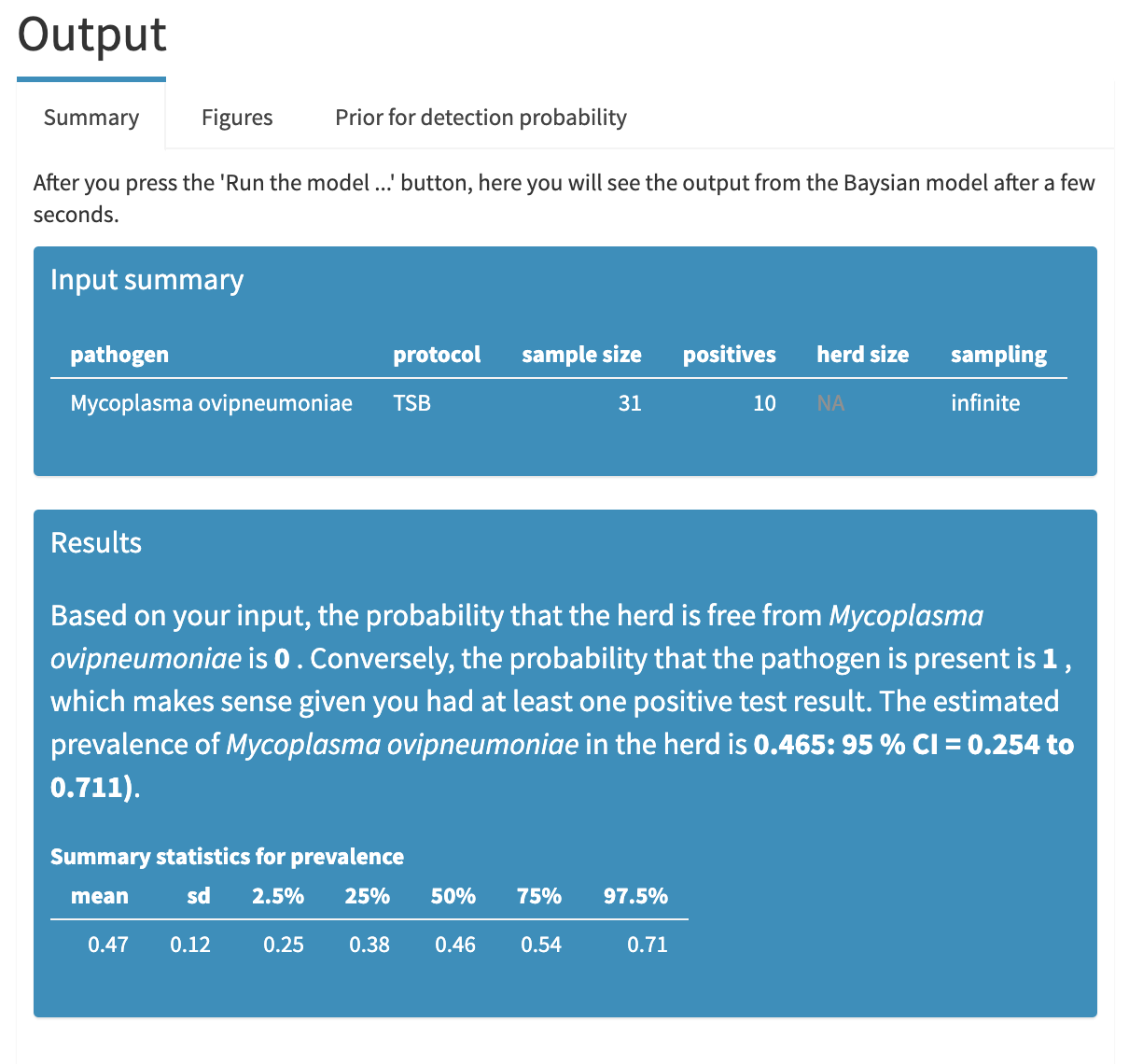

Supplement: S3 Fig — In this specific example, 10 out of 31 animals in the Taylor-Hilgard herd (2016–2017) tested positive for Mycoplasma ovipneumoniae using the TSB-PCR protocol with 1 swab per animal. (TIF) [file pone.0237309.s003.tif]

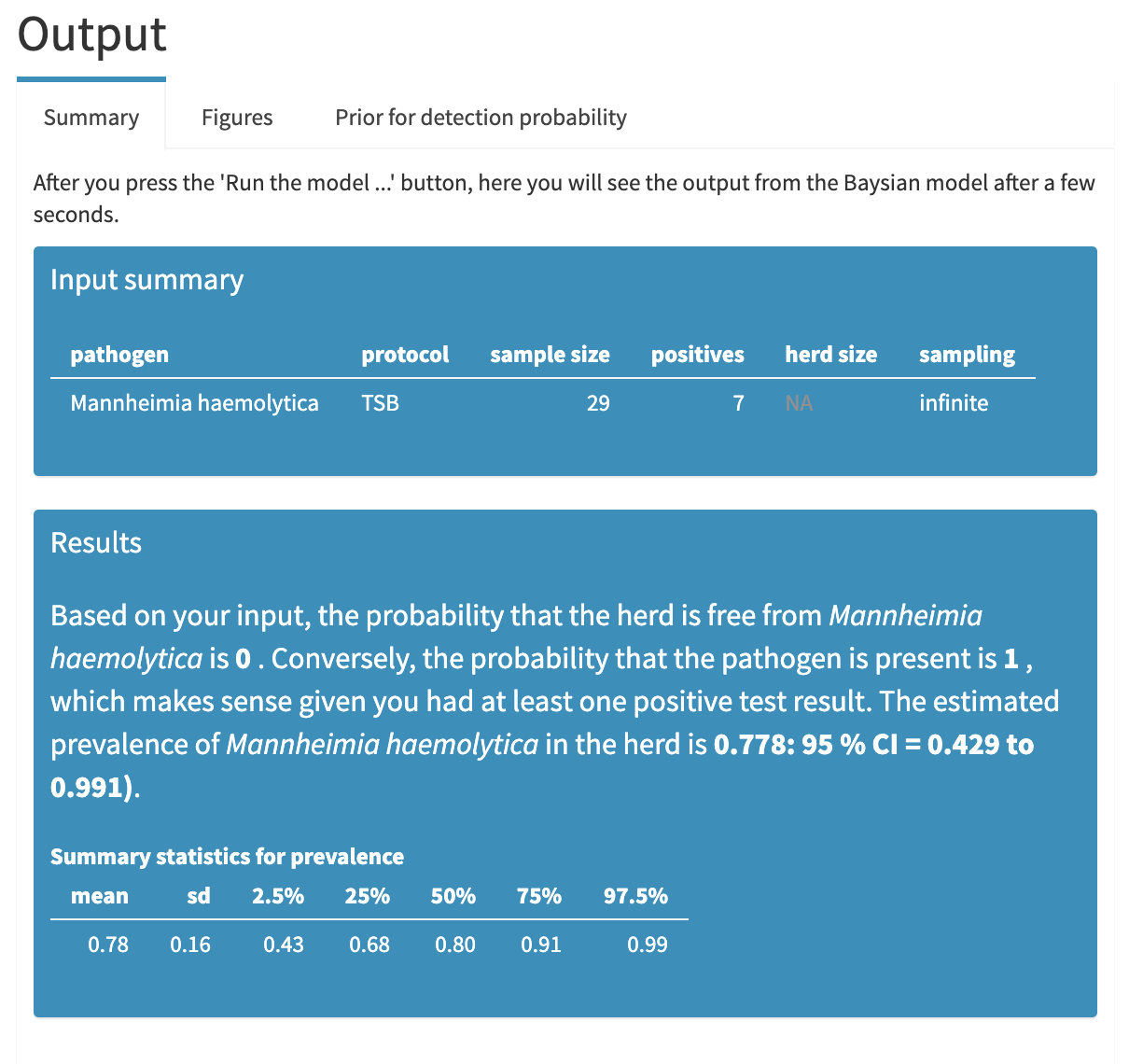

Supplement: S4 Fig — In this specific example, 7 out of 29 animals in the Taylor-Hilgard herd (2013–2014) tested positive for Mannheimia haemolytica using the TSB-culture protocol with 1 swab per animal. (TIF) [file pone.0237309.s004.tif]

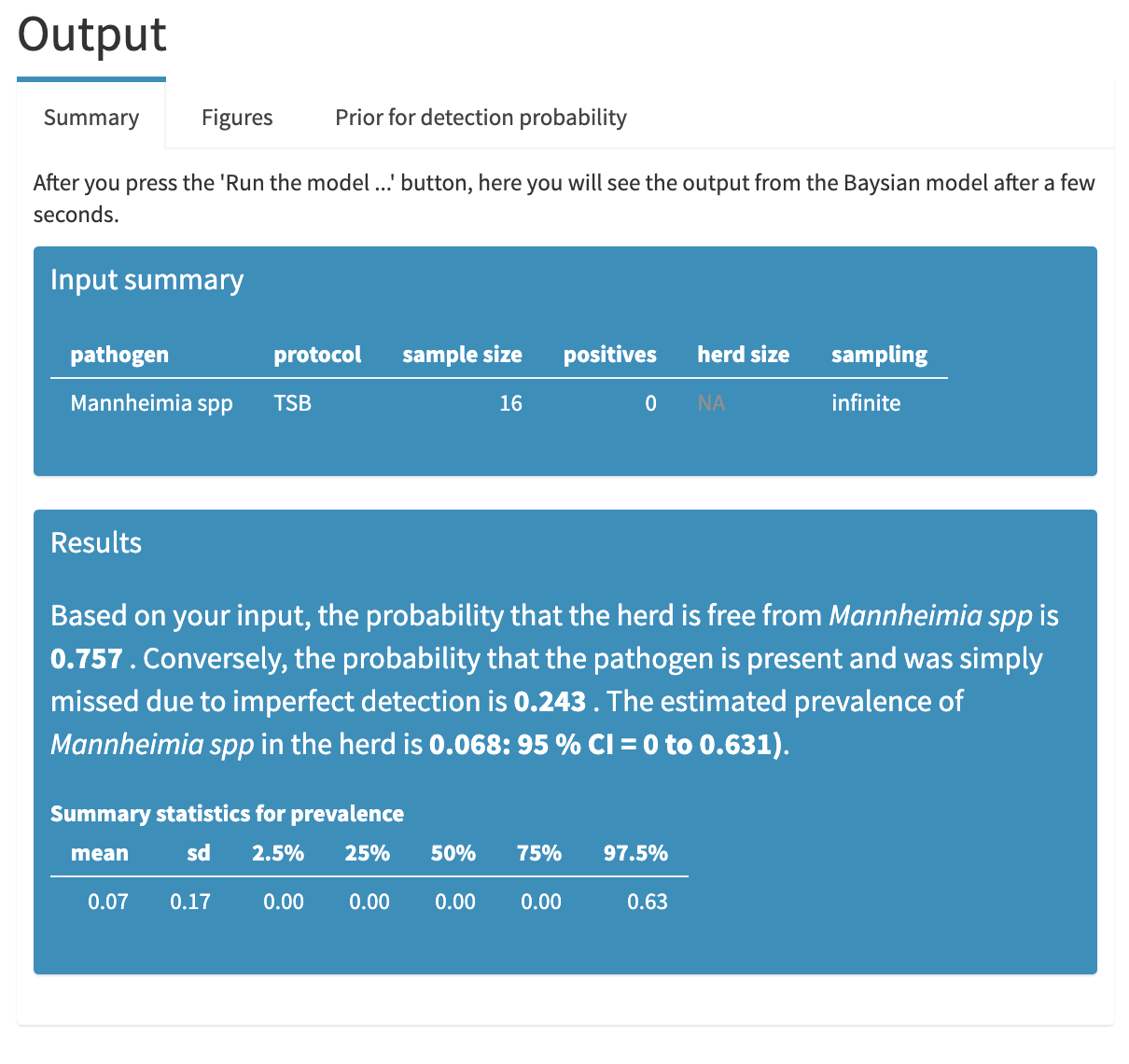

Supplement: S5 Fig — In this specific example, 0 out of 16 animals in the Highlands herd (2015–2016) tested positive for Mannheimia spp. using the TSB-culture protocol with 1 swab per animal. (TIF) [file pone.0237309.s005.tif]

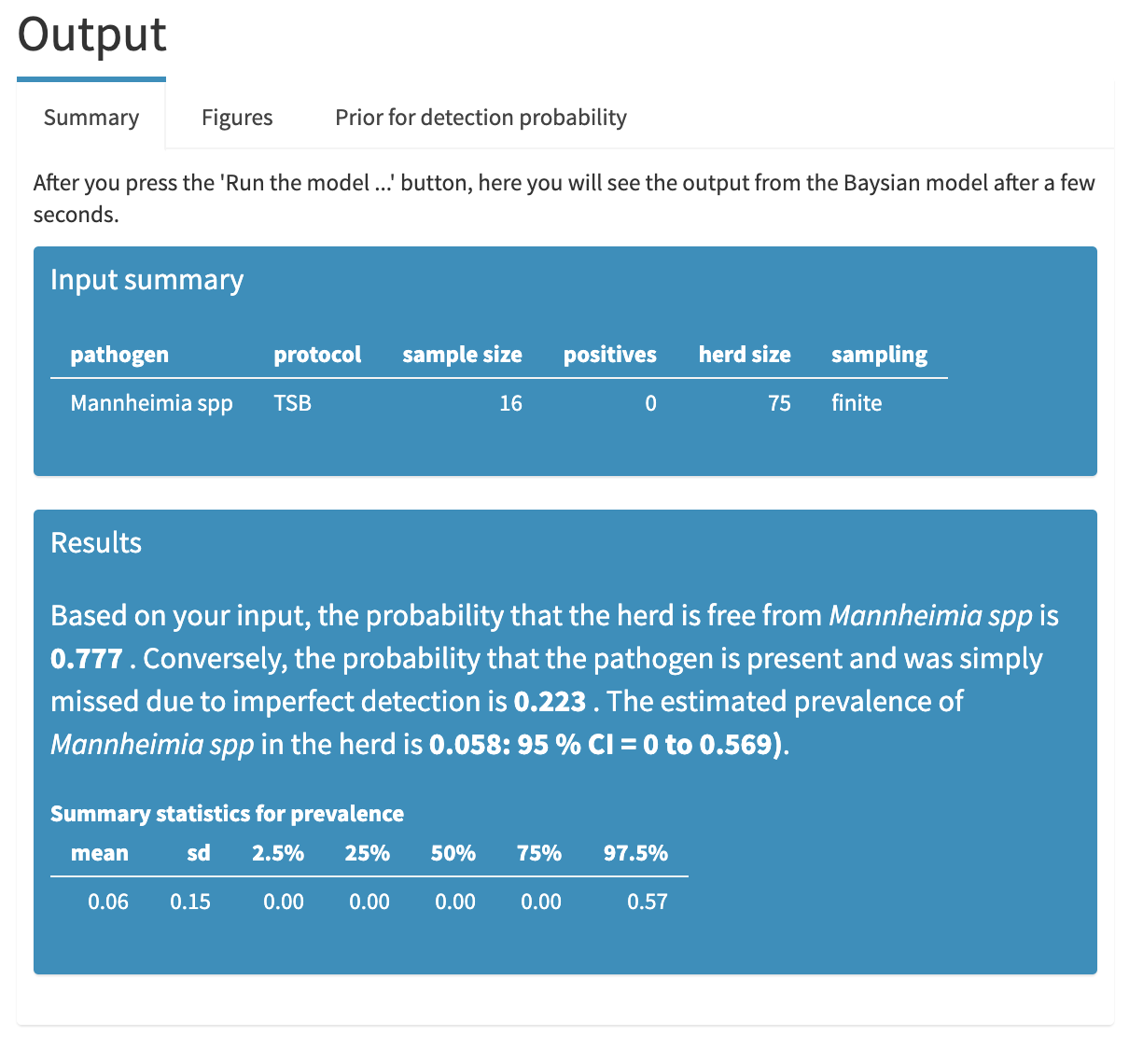

Supplement: S6 Fig — In this specific example, 0 out of 16 animals in the Highlands herd (2015–2016) tested positive for Mannheimia spp. using the TSB-culture protocol with 1 swab per animal. (TIF) [file pone.0237309.s006.tif]
