# Supplementary material for: How sure are you? A web-based application to confront imperfect detection of respiratory pathogens in bighorn sheep
Source: PLoS One. 2020 Sep 8;15(9):e0237309. doi: 10.1371/journal.pone.0237309 (PMC7478830; doi:10.1371/journal.pone.0237309)
Supplement: S1 Table — (DOCX) [file pone.0237309.s007.docx]

S1 Table

| **Pathogen** | **Protocol** | **Description** |
| --- | --- | --- |
| *Mycoplasma ovipneumoniae* | TSB-PCR^1^ | A nasal swab was collected by inserting sterile polyester applicators (Puritan#25-806 1PD, Guilford, ME, USA) 8-12 cm into the nasal cavity and slowly rotating the shaft and placed immediately into a vial of tryptic soy broth (TSB). Samples were frozen as soon as possible and shipped overnight on dry ice to Washington Animal Disease Diagnostic Laboratory (WADDL) for *Mycoplasma ovipneumoniae* PCR testing. |
| *Mannheimia haemolytica* | TSB-culture^2^ | A tonsil swab was collected using sterile polyester applicators (Puritan#25-806 1PD, Guilford, ME, USA) applied to the tonsilar crypts and the outer tonsil surface. The applicator was placed immediately into a vial of tryptic soy broth with 15% glycerol (TSB; Hardy Diagnostics, Santa Maria, California, USA). Samples were frozen as soon as possible and shipped overnight on dry ice to Washington Animal Disease Diagnostic Laboratory (WADDL) for *Pasteurellaceae* culture following the lab’s standard operating procedures. Swabs remained frozen at WADDL until they were plated by diagnosticians. |
