# Supplementary material for: How sure are you? A web-based application to confront imperfect detection of respiratory pathogens in bighorn sheep
Source: PLoS One. 2020 Sep 8;15(9):e0237309. doi: 10.1371/journal.pone.0237309 (PMC7478830; doi:10.1371/journal.pone.0237309)
Supplement: Attachment — Submitted filename: Pathogens_run.pdf [file pone.0237309.s008.pdf]

Imperfect detection

This section is more general in that it allows you to vary many of the probabilities that underlie the other two sections. As a result, it is also more technical, and includes information on prior distributions and raw output from the Bayesian model that the other two sections do not.

Specifics of the sampling design

Are you assuming a finite or infinite population?

- (●) finite; sample size > 10% population
- ( ) infinite; sample size < 10% population

Select the number of animals sampled

5

Select the number of individual swabs per animal

3

Select the number of individuals with a positive test

1

Herd size

10

Prior for the presence of the pathogen

Set equal to 0.5 if you have no information.

Pathogen presence

0 0.5 1

0 0.1 0.2 0.3 0.4 0.5 0.6 0.7 0.8 0.9 1

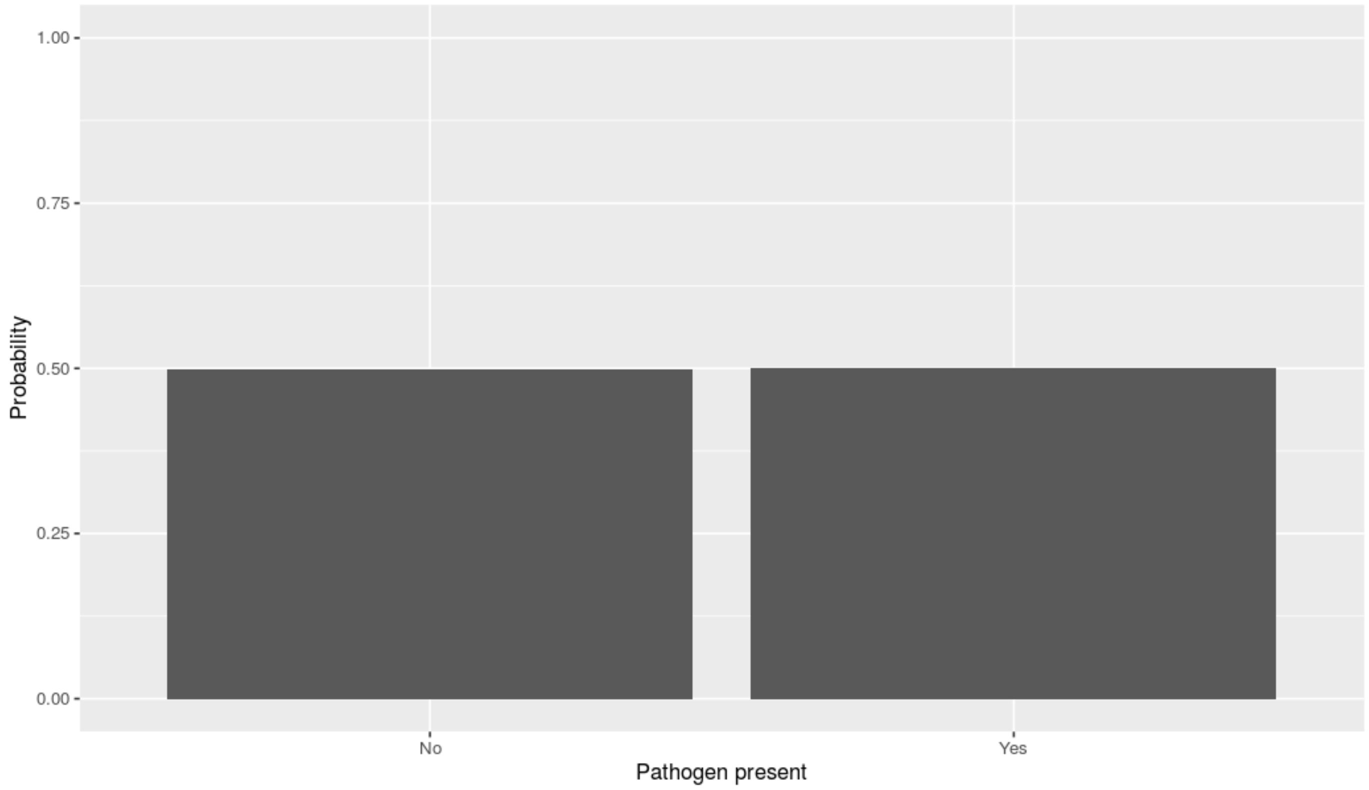

Prior for the prevalence of the pathogen

Set both equal to 1 if you have no information

alpha for prevalence

3

beta for prevalence

1

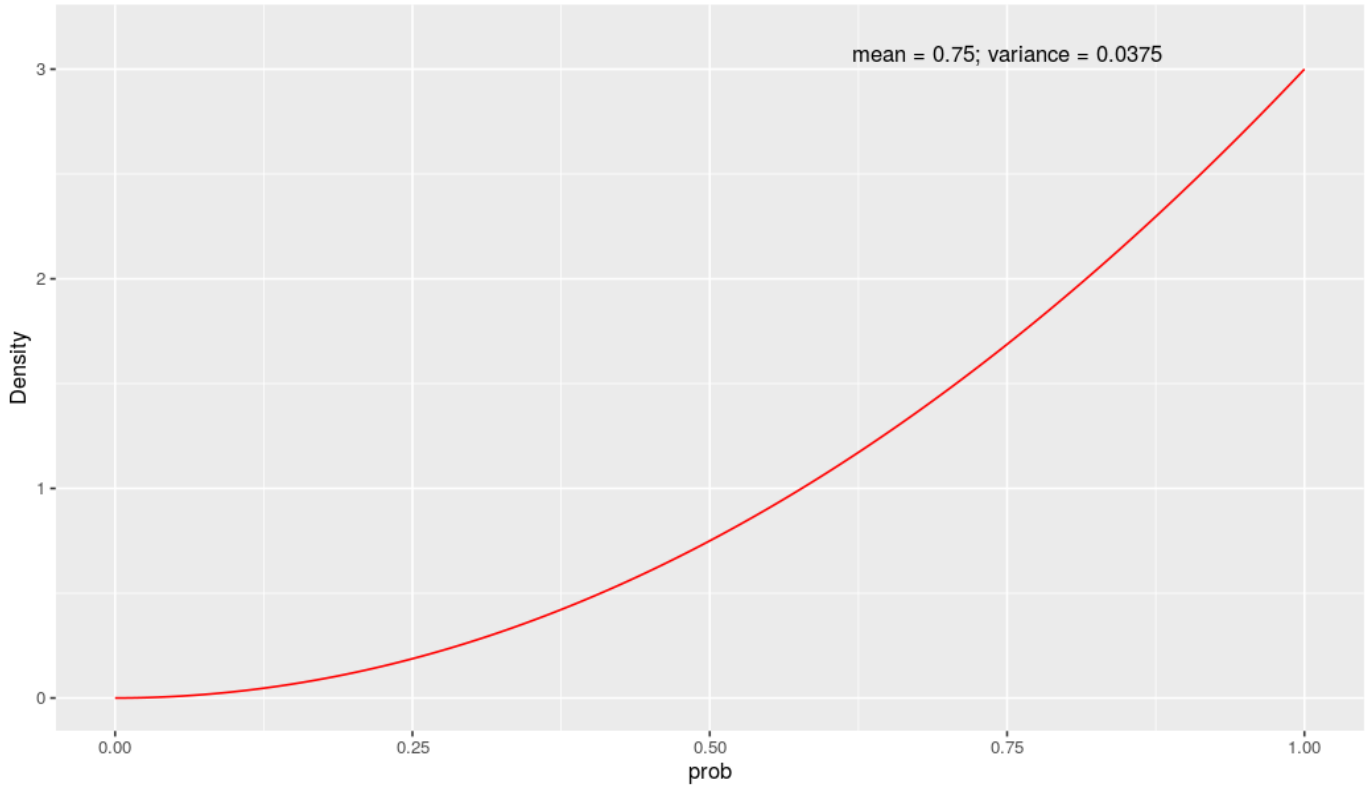

Prior for the detection probability

Set both equal to 1 if you have no information

alpha for detection

20

beta for detection

1

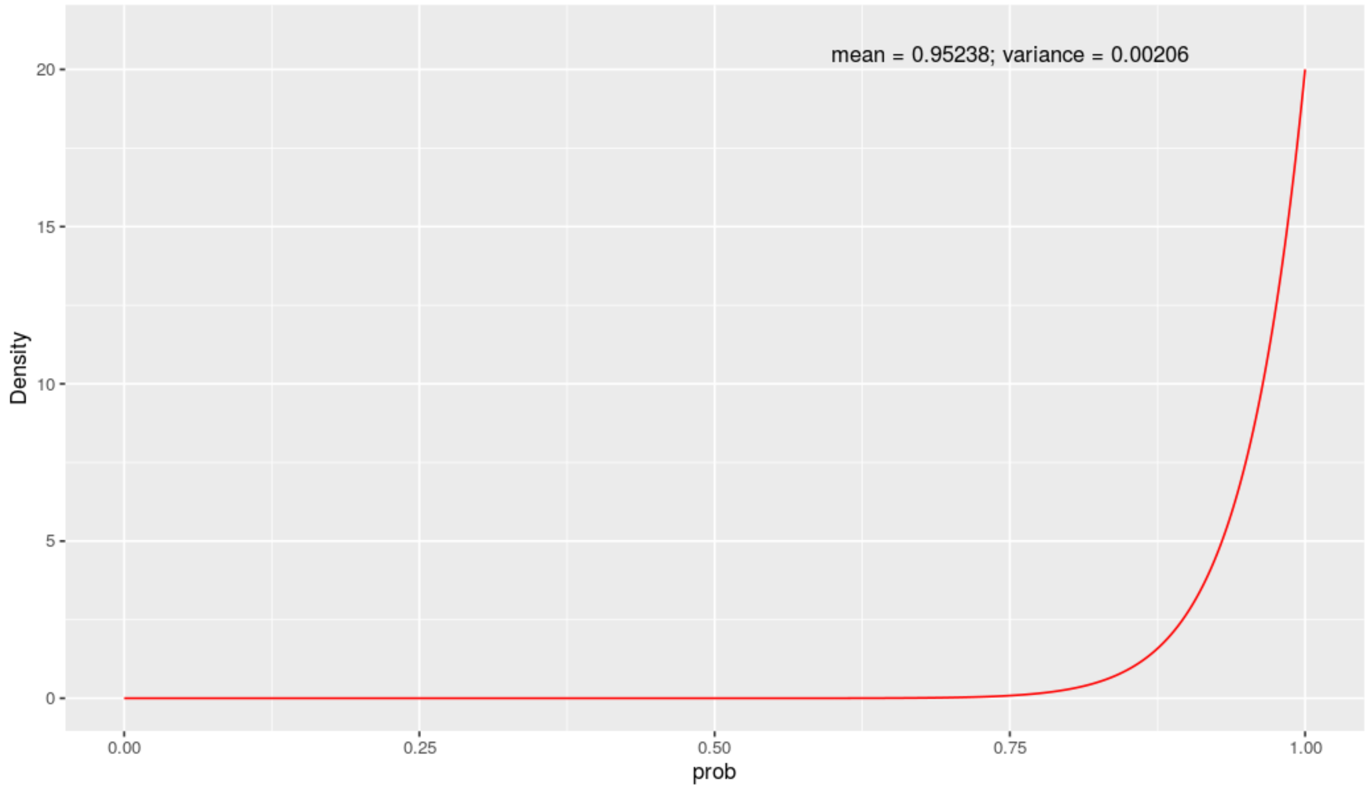

Run model (wait a few seconds)

Model results

The results of the model indicated ...

Output from JAGS

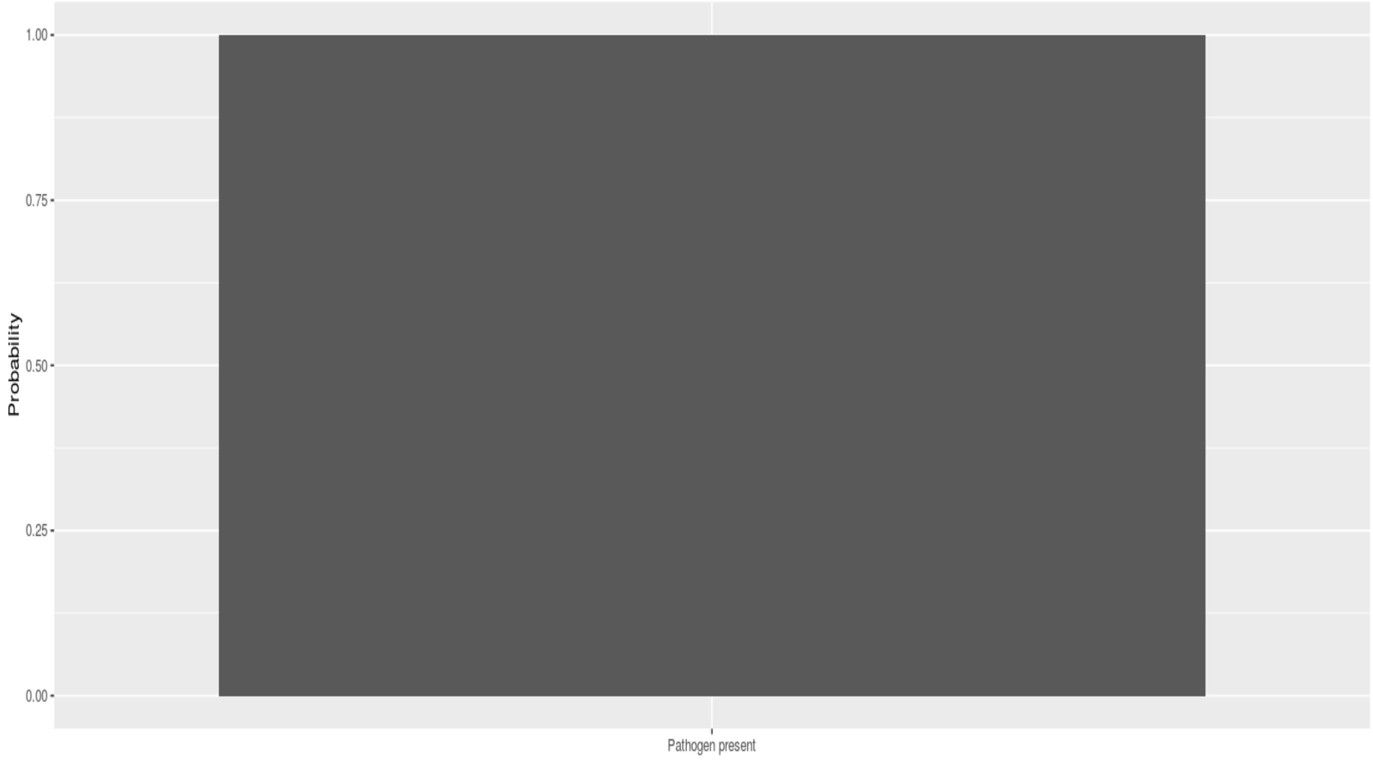

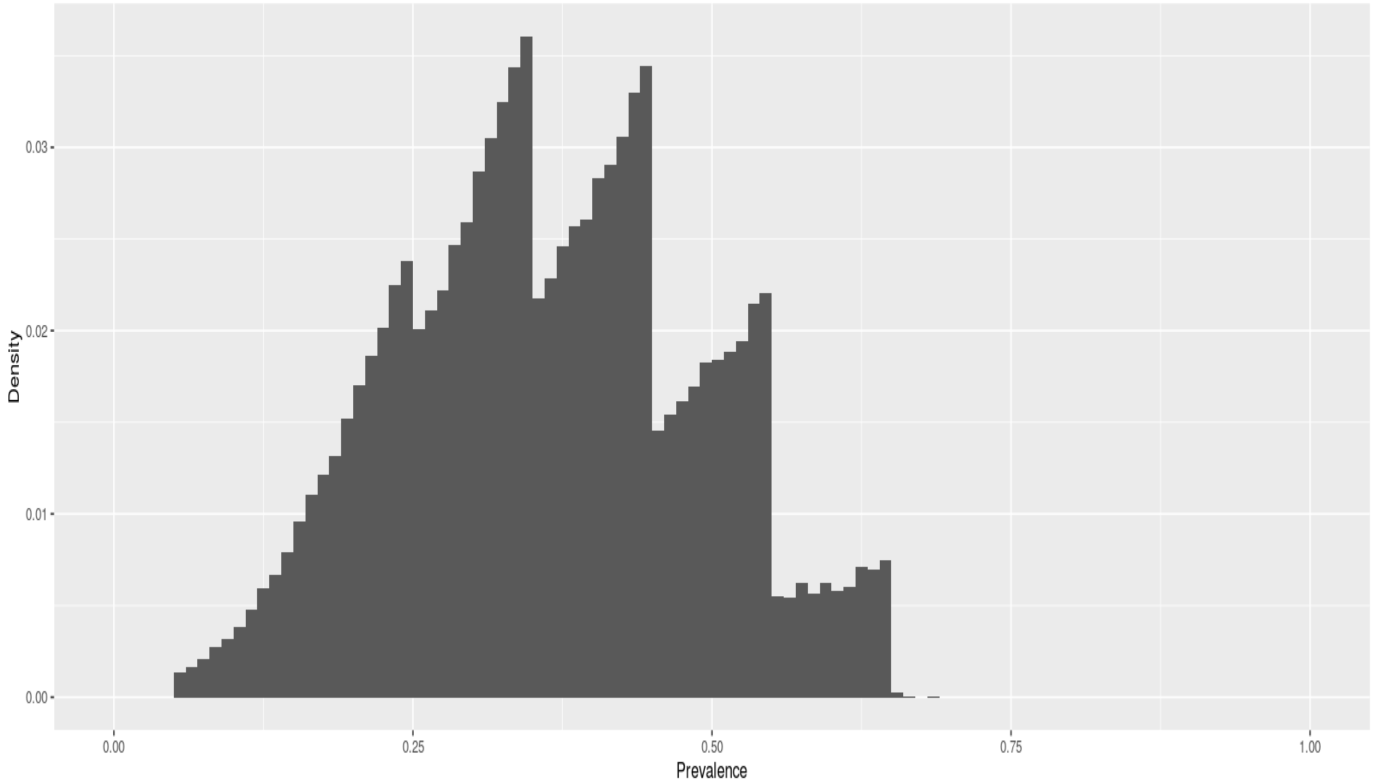

parameter	Mean	SD	Lower95	Median	Upper95
Free from pathogen	0.00	NA	NA	NA	NA
True prevalence	0.36	0.12	0.13	0.36	0.61
