# Supplementary material for: How sure are you? A web-based application to confront imperfect detection of respiratory pathogens in bighorn sheep
Source: PLoS One. 2020 Sep 8;15(9):e0237309. doi: 10.1371/journal.pone.0237309 (PMC7478830; doi:10.1371/journal.pone.0237309)
Supplement: Attachment — Submitted filename: Response to Reviewers_24281R2.docx [file pone.0237309.s009.docx]

**Responses to general/major comments (comments are italicized, responses are in normal font, response line numbers refer to revised manuscript):**

**Academic Editor:**

*Also, it would be important to provide the complete code of the application as this would benefit readers with partial knowledge of approach used and would likely increase the impact of the manuscript.*

We agree with the academic editor and have included a new section on the “Model examples” tab in the shiny application itself where we provide complete code for how to use these models given a set of detection results and the prior information from Butler et al. 2018.

**First reviewer**

*- Line 10 “Our primary objective was to develop an easy-to-use and accessible web-based Shiny application that estimates the probability (with associated uncertainty) that a respiratory pathogen is present in a herd and its prevalence” – I suggest adding “given imperfect detection” at the end of the sentence.*

We agree this is an improvement, and have added the text at the end of the sentence on lines 8 to 10 to now read:

“Our primary objective was to develop an easy-to-use and accessible web-based Shiny application that estimates the probability (with associated uncertainty) that a respiratory pathogen is present in a herd and its prevalence given imperfect detection.”

*- Line 13 “We found that one population with no detections of Mycoplasma ovipneumoniae (PCR assay) still had an 8% probability of the pathogen being present in the herd” – I suggest adding “For instance,” to avoid readers thinking that the authors means that no detection systematically indicate a true prevalence of 8%.*

We agree this is an improvement, and have added the text at the beginning of the sentence on lines 13 to 15 to now read:

“For instance, one population with no detections of *Mycoplasma ovipneumoniae* (PCR assay) still had an 6% probability of the pathogen being present in the herd”

*- Between lines 13-22, the structure “We found…” is used three times. I suggest to reword to make it more pleasant to read.*

We have re-written lines 13 to 22 to avoid the repetition and remove “we found” in three of the four instances. The lines now read:

“For instance, one population with no detections of *Mycoplasma ovipneumoniae* (PCR assay) still had an 6% probability of the pathogen being present in the herd. Similarly, the apparent prevalence (0.32) of *M. ovipneumoniae* in another herd was a substantial underestimate of estimated true prevalence (0.46: 95% CI = [0.25, 0.71]). The negative bias of naïve prevalence increased as the probability of detection of testing protocols worsened such that the apparent prevalence of *Mannheimia* *haemolytica* (culture assay) in a herd (0.24) was less than one third that of estimated true prevalence (0.78: 95% CI = [0.43, 0.99]). We found a small difference in the estimates of the probability that *Mannheimia spp.* (culture assay) was present in one herd between the binomial sampling approach (0.24) and the hypergeometric approach (0.22)”

*- Line 70 “However, test sensitivity for wild hosts can be estimated using occupancy models, which is a flexible and straightforward method of evaluating test performance while incorporating multiple layers of uncertainty in a hierarchical modeling framework [20,29].” I suggest using “approximated” instead of “estimated” to make it clearer that it is almost impossible to determine with certainty the true sensitivity of a test, especially in wild populations. Idem line 73. I overall think that this part of the text (lines 69-75) is too optimistic (see my previous review) and that the authors should be more careful. For instance, I would suggest something like “Although estimating the true sensitivity of a test can be challenging (Enøe et al. 2006), especially in wild populations, it can be approximated…”, then explain that even if not perfect, it is still better to correct prevalence estimates based on this approximated sensitivity than not to correct at all.*

We appreciate the reviewer taking the time to further elaborate on this key point from the first review, and we acknowledge the importance of being more careful with our language. We have further revised the text on lines 68 to 77 to make it more clear that this method remains an approximation of true sensitivity. These lines now read:

“For populations of wild hosts for which there are few gold-standard reference tests and in which the true disease state of individuals are not known, estimating the sensitivities and specificities of diagnostic tests can be challenging [28]. However, test sensitivity for wild hosts can be approximated using occupancy models, which is a flexible and straightforward method of evaluating test performance while incorporating multiple layers of uncertainty in a hierarchical modeling framework [20,29]. Although this is only an approximation to the true, unknown, test sensitivity, it is still an improvement to correct prevalence estimates using this approximated sensitivity. The relative ease with which detection probability of a diagnostic testing protocol can be approximated, coupled to the consequences to inference for failing to do so, suggests that explicitly accounting for imperfect detection should be the paradigm for wildlife disease monitoring programs [19,23].”

*- Line 86 – No need to specify that (M. ovipneumoniae) stands for Mycoplasma ovipneumoniae. Such abbreviations are standard.*

Revised on line 88.

*- Please set the example and provide the complete codes for the models. This will be useful for people knowing how to code but not yet completely confident with JAGS or Bayesian statistics in general and increase the impacts of your study.*

We agree with this point that was also raised by the Academic Editor. We have included a complete example on the shiny application itself (under the “Model Examples” tab) that illustrates how to: 1) incorporate the information on detection priors from Butler et al., 2) specify the information on sampling design and results (number of swabs and detections), 3) run the models using the runjags package as an interface to the JAGS program, and 4) access the results.

*- Lines 204-205 – I would suggest “note that additional Pasteurellaceae/protocol combinations are available in the online application” to sound more positive.*

Revised on lines 204 to 205, which now reads:

“(although note that additional *Pasteurellaceae*/protocol combinations are available in the online application). ”

*- Line 213 – Space missing after “(Table 1).”*

Revised on line 214.

*- Lines 207-220 – You could make this part more fluent by saying something like: “Duplicated swabs [please indicate types of swabs: nasal?) were collected and stored in tryptic soy broth (TSB) from [add information about the designs: adults/juveniles, males/females/both, during breeding season/during an outbreak/over X breeding seasons…]. The swabs were analyzed by PCR/culture to detect…”*

Rather than repeat these results from the previous work upon which our current manuscript is based, we have highlighted on line 207 that the sampling details are available in this previous body of research. However, we have added “nasal” to be clear that nasal swabs were used during sampling, as per the reviewer’s request. Lines 204-222 now read:

“Here, we use four examples of pathogen testing from recently published research on bighorn sheep respiratory pathogens [19,35] to illustrate the consequences of imperfect detection for estimating true prevalence (Table 1) (although note that additional *Pasteurellaceae*/protocol combinations are available in the online application). We chose three herds from Montana (Petty Creek, Taylor-Hilgard herd, and Highlands) (Fig 1) that were tested for a diverse group of pathogens using multiple testing protocols that can best illustrate the consequences of imperfect detection (details available in [19,35]). The primary testing protocol in this study for *M. ovipneumoniae* in bighorn sheep utilized tryptic soy broth (TSB) as a transport media with a PCR assay (named the “TSB-PCR Protocol”). Based on nasal swab samples tested by PCR and analyzed in an occupancy-modeling framework, this protocol has a modestly high probability of detection ($\hat{\mathrm{detection}}=0.72, [95\% credible interval, \mathrm{CI}=0.62-0.81]$) [19,35]. Using this protocol with two nasal swabs per animal, the apparent prevalence of *M. ovipneumoniae* in the Petty Creek herd in the winter of 2015-2016 was 0, and 0.32 for the Taylor-Hilgard herd using a single nasal swab in the winter of 2016-2017 (Table 1). In contrast to the TSB-PCR protocol for *M. ovipneumoniae*, the primary testing protocol for *Mannheimia haemolytica* (a *Pasteurellaceae*) (tryptic soy broth with a culture assay, named “TSB-culture”) based on nasal swab samples has a substantially lower probability of detection ($\hat{\mathrm{detection}}=0.24, [95\% \mathrm{CI}=0.15-0.32]$) [19,35]. Using this protocol, the apparent prevalence of *Mannheimia haemolytica* in the Taylor-Hilgard herd using a single nasal swab in the winter of 2013-2014 was 0.24. Finally, the apparent prevalence of *Mannheimia spp.* (a *Pasteurellaceae*) in the Highlands herd in the winter of 2015-2016 using the TSB-culture testing protocol with two nasal swabs per animal was 0 ($\hat{\mathrm{detection}}=0.09, [95\% \mathrm{CI}= 0.07-0.12]$) [19,35]”

*- Line 216 – “based a single swab”. Is “on” missing?*

Yes it was. We have revised that section as above.

*- Lines 215-216 – “based a single swab has a substantially lower probability of detection (detection ̂ = 0.24, [95% CI = 0.12 - 0.40])” this sentence is confusing. I understand from it that detection probability was estimated from a sampling design including only one swab per individual.*

Revised on lines 216 to 219 to now read:

“In contrast to the TSB-PCR protocol for *M. ovipneumoniae*, the primary testing protocol for *Mannheimia haemolytica* (a *Pasteurellaceae*) (tryptic soy broth with a culture assay, named “TSB-culture”) based on nasal swab samples has a substantially lower probability of detection ($\hat{\mathrm{detection}}=0.24, [95\% \mathrm{CI}=0.15-0.32]$) [19,35].”

*- In the app, when looking at the prior plot from the outputs of the prevalence, the title of the plot does not fit (half is missing), same for the prevalence figure although the title is on two lines.*

We have revised the application to include a line-break in the plot titles.

*- Line 244 – I would add a mention of the third component, which allows to explore the impact of detection probability on prevalence estimation. Idem in Figure 2 legend.*

Revised on lines 244 to 247 and in the Figure 2 legend.

*- Line 259 – “combination[19,35]” missing space before references. Idem line 262.*

Revised on lines 263 and 266.

*- I did not realized when I reviewed the previous version of the manuscript but instead of a clear unique probability of pathogen presence, does not the model report a posterior distribution for this probability? If yes, would not it be more reasonable to report a violin plot instead of a bar plot? Or a histogram like you do for the prevalence? In addition, the two bars are confusing as they are redundant (right?). I would suggest to remove one of them.*

The reviewer was correct in the review of the previous submission that there is a single, unique probability of pathogen presence. For each iteration of the MCMC algorithm, the model is imputing the presence of the pathogen as either a 1 (pathogen present), or a 0 (pathogen absent). Over the course of the entire model run, the probability of pathogen presence is the proportion of the iterations where the pathogen was estimated to be present, i.e., there is no posterior distribution for the probability of pathogen presence due to it being derived from the posterior distribution of pathogen state.

We agree that the two bars are redundant, and have removed the bar for “Pathogen present”.

*- Line 273 – “a non-zero probability that the pathogen was in the herd and simply missed”. Does your model only account for the fact that pathogens could be missed in the sampled individuals (test sensitivity), or also for the fact that you can have missed (not sampled) infected individuals? In any case, this sentence should state “in the sampled individuals” as you specifically talk about detection probability here or the sentence should be reworded.*

This model accounts for both: the uncertainty in the final estimates of prevalence incorporates both the uncertainty in test sensitivity (inflating apparent prevalence by test sensitivity), as well as uncertainty due to sample size.

Reworded as suggested on lines 275-278 to now read:

“The apparent prevalence in this case is 0%; however, the very low probability of detection for this pathogen/protocol combination implies a non-zero probability that the pathogen was in the herd and simply missed in the sampled individuals, with significant resulting uncertainty in what the true prevalence was [95% CI = 0.0 – 0.81] (Fig 4).”

*- Line 294-295 – “0 out of 16 animals with a positive test”. I suggest “0 positive test out of16 tested animals”.*

Revised on lines 299-300.

*- Line 205 – Remove “TSB” as this is true for any protocol and TSB is the name of the storage medium, not the testing protocol (PCR or culture would be more correct). Idem lines 304 and 312.*

Revised on lines 301, 309, 317, and 325 (we assumed line 301 was the intended target, not line 205), as well as in Supplementary Figure captions.

*- Lines 307-309 – “The uncertainty associated with this prevalence estimate (and all such estimates based on small samples with imperfect and uncertain detection) is substantial and may render this estimate of little value for understanding disease etiology”. True but this is mostly due to small sample size, isn’t it? If you reported confidence intervals around the apparent prevalence (e.g., Clopper-Pearson confidence interval) that would help the reader noticing that uncertainty is not negligible even if detection probability was considered to be one.*

We thank the reviewer for this good suggestion, and have reported credible intervals for apparent prevalence both in the text and in the application itself.

*- Please state somewhere that CI stands for credible interval (as by default I think most people will assume it stands for confidence interval), right?*

We have included this information on line 211 to make clear that we mean “credible interval” for CI.

*- Line 368 – Please use “more accurate” instead of “unbiased”.*

Revised on line 373.

*- For the imperfect detection part of the app, I tried with the default conditions but 3 swabs and 3 positive individuals and got “An error has occurred. Check your logs or contact the app author for clarification.”. This was, I assume, due to positive > sampled (I put random numbers) but a more explicit error message could be printed (e.g. “Number of positive individuals cannot be superior to number of sampled individuals”.*

This is an excellent suggestion, and we have included more informative error messages in the application as the reviewer suggests.

*- The posterior distributions I obtain show weird steps (see attached PDF). Is it normal? Where is that coming from?*

This is an expected result that is a combination of two factors: hypergeometric sampling and the informed prior you used for prevalence in this example. There are a discrete number of animals that host the pathogen in any population, a number that is estimated as the product of true prevalence and the size of the population. The rounding that is required to use the hypergeometric distribution is what gives that distribution the steps you note. The “pull to the side” on the steps is a result of you using an informed prior on prevalence that “wants” to pull those estimates to the right.

*- Line 387 – Culture also allows “current detection of the bacteria”. I would suggest “characterization of the infection status at the moment of sampling” (the bacteria is currently present) as I am not sure “current detection of the bacteria” is correct as “current” defines detection here, not the bacteria presence*

Revised as the reviewer suggests on line 392.

*.*
*- Lines 400-401 – I think a promising avenue for individual status characterization is the combination of multiple tests and probabilistic approaches, as illustrated in Buzdugan et al. 2017. Mentioning this explicitly would make this perspective more concrete.*

We thank the reviewer for this suggestion, and have revised lines 405 to 407 to now read:

“Future work is required to address how they may translate to the individual level if prevalence is related to individual characteristics, and a promising direction takes advantage of a multiple-testing protocols using a longitudinal design in a probabilistic framework [43].”
